# Dilemmas in the usage of community-based home care and smart home care services for older adults among family caregivers of older adults with mild cognitive impairment (MCI): a qualitative study

**DOI:** 10.3389/fpubh.2025.1694462

**Published:** 2025-12-23

**Authors:** Yulu Chen, Ni Li, Xiaoling Bai, Yan Jiang, Bingxue Tang, Shan Huang, Juan Wu

**Affiliations:** 1 School of Nursing, Chongqing University of Humanities, Science and Technology, Hechuan, Chongqing, China; 2 Department of Nursing, Guizhou Provincial Rehabilitation Hospital, Guiyang, Guizhou, China; 3 School of Nursing, Guizhou College of Nursing, Guiyang, Guizhou, China; 4 Department of Psychiatry, Chongqing Mental Health Center, Liangjiang New Area District, Chongqing, China; 5 Department of Science and Technology, Guizhou College of Nursing, Guiyang, Guizhou, China; 6 Department of Nursing, Shanghai Children’s Medical Center Guizhou Hospital, Shanghai Jiao Tong University School of Medicine, Guiyang, Guizhou, China

**Keywords:** older adults with mild cognitive impairment (MCI), family caregivers, community-based home care, smart older adults’ care service use, caregiving challenges, qualitative research

## Abstract

**Objective:**

To explore the caregiving challenges encountered by family caregivers of older adults with mild cognitive impairment (MCI), and to proffer recommendations for optimizing older adults’ care services in China and enhancing the quality of long-term care for older adults with MCI.

**Methods:**

A phenomenological approach was employed to conduct semi-structured interviews with 20 family caregivers of older adults with mild cognitive impairment (MCI). This study employed the Colaizzi analytical method and systematically organized and analyzed the data using NVivo 12.0 software.

**Results:**

Three core themes and nine sub-themes were identified: (1) Inadequate adaptability of external support systems (structural gaps in community-based home care policies and evaluation frameworks, shortages in caregiving knowledge and skills provision, sustainability challenges in financial support); (2) Dual social and emotional burdens faced by caregivers (disruption of social networks and isolation, accumulation of negative emotional experiences and burnout, moral conflicts over family perceptions and responsibility allocation); (3) Obstacles in smart older adults’ care technology use (Privacy concerns from smart monitoring systems, the digital divide, lack of emotional connectivity in human-computer interaction).

**Conclusion:**

Relevant departments should focus on the burdens and challenges family caregivers face in caring for older adult individuals with mild cognitive impairment. This will enable them to develop further diverse community-based home and smart older adults’ care services, ultimately promoting the high-quality development of China’s older adults’ care sector.

## 1 Introduction

Mild cognitive impairment (MCI) represents a transitional state between normal cognitive aging and Alzheimer’s disease (1). In China, the population of older adults with MCI has surpassed 38 million, ranking first globally (2). The condition is often insidious: in its early stages, it presents primarily as subtle memory deficits, yet most cases gradually progress to severe cognitive impairment and functional disabilities within several years (3). Given that cognitive impairment is irreversible and lacks specific medical interventions, its diagnosis imposes substantial economic, physical, and psychological burdens on both patients and their families (4). The majority of older adults with MCI reside in community-based family settings (5). However, with the evolution of family structures, traditional home-care models increasingly struggle to address the escalating pressures of aging (6). Family caregivers must continually monitor the physical condition of older adults and manage emergencies arising from cognitive decline and functional impairments (7). These responsibilities can induce anxiety, fatigue, and helplessness, potentially disrupting caregivers’ work and daily lives (8). Prolonged high-stress conditions among family caregivers may not only compromise the quality of care provided to older adults but also trigger social issues, such as intensified family conflicts and inefficient utilization of medical resources (9). Community-based home care and smart aging technologies have offered new avenues to address aging challenges (10), yet family caregivers of older adults with MCI face numerous practical obstacles (11). Understanding these caregiving challenges is crucial for tailoring older adults’ care services and alleviating the burden on caregivers. This study employs qualitative research to interview family caregivers of older adults with MCI, analyze existing gaps in older adults’ care services, and provide insights for optimizing China’s older adults care sector.

## 2 Methods

### 2.1 Design and sample

This study employed purposive sampling and was conducted in communities of Guiyang City from January to June 2024. All researchers had received systematic training in qualitative research methodologies. Inclusion criteria were as follows: (1) Immediate family members of older adults with mild cognitive impairment (MCI) serving as primary caregivers; (2) Individuals assuming primary caregiving responsibilities for MCI patients, providing continuous care for ≥3 months, and not receiving monetary compensation for their labor; (3) Aged ≥18 years; (4) Mentally alert individuals capable of unimpeded verbal communication; (5) Voluntary participants who signed the informed consent form. Exclusion criteria: Caregivers whose care recipients resided in nursing homes.

### 2.2 Participant characteristics and ethical considerations

Following the principle of maximum diversity in sampling, caregivers exhibiting significant variations in gender, age, marital status, place of residence, educational attainment, and relationship to the care recipient were selected for inclusion in the study. The sample size was determined based on data saturation, with analysis concluding when no new themes emerged. A total of 20 family caregivers were recruited, each assigned a participant code (P1 to P20) according to the interview sequence. Detailed demographic characteristics are presented in Table 1. This study was approved by the Ethics Committee of Guizhou Nursing Vocational and Technical College (Approval No: gzhlllscb2022-1203).

**Table 1:** Detailed demographic data of the participants.

Number	Gender	Age (years)	Marital status	Residence	Education level	Relationship to care recipient
P1	Male	41	Married	Urban	High school	Father and son
P2	Female	50	Married	Urban	Junior high school	Father-daughter
P3	Female	69	Married	Urban	Junior high	Married
P4	Male	45	Married	Urban	Bachelor’s degree	Mother and child
P5	Female	43	Married	Urban	High school	Father-daughter
P6	Male	49	Married	Rural	Junior high	Mother and son
P7	Male	73	Married	Urban	Bachelor’s degree	Married
P8	Male	49	Married	Rural	College	Mother and child
P9	Female	51	Married	Urban	Elementary school	Mother and daughter
P10	Female	42	Married	Urban	High school	Mother and daughter
P11	Male	46	Married	Rural	High school	Father and son
P12	Male	47	Divorced	Urban	College	Father and son
P13	Female	35	Divorced	Urban	College	Father-daughter
P14	Male	45	Married	Urban	Elementary school	Father and son
P15	Female	38	Married	Urban	College	Mother and daughter
P16	Female	55	Divorced	Township	Elementary school	Mother and daughter
P17	Male	40	Married	Urban	Junior high	Mother and child
P18	Female	48	Divorced	Urban	High school	Mother and daughter
P19	Male	38	Married	Urban	Bachelor’s degree	Father and son
P20	Female	49	Divorced	Urban	High school	Mother and daughter

### 2.3 Interview outline

This study employs a phenomenological research approach within the qualitative research framework, utilizing semi-structured interviews to explore the caregiving challenges faced by family caregivers of older adults with mild cognitive impairment (MCI) in home care settings and to analyze the current issues in community-based older adults’ care services. Based on the literature review and expert consultation, a preliminary interview outline was developed. Five caregivers were interviewed in a pre-interview phase, and the final interview outline was formulated based on the interview findings and participants’ feedback. The main content of the interview outline includes:

1. Have you accessed community-based home care services? Do these services align with your actual care needs?
2. What are your perceptions and experiences regarding community-based home care services?
3. During the caregiving period, what significant changes have occurred in your personal life?
4. What difficulties have you encountered in caring for older adults with MCI (e.g., lack of caregiving skills, emotional challenges, financial burdens)?
5. What specific emotions or feelings have emerged during the caregiving process? How have these emotions influenced your daily life or caregiving practice?
6. Have you used or considered using intelligent older adults’ care technologies or services for caregiving? What are your attitudes toward them, and how was your experience with their application?

### 2.4 Data collection methods

Data collection employed face-to-face, semi-structured, in-depth interviews. Interviews were conducted one-on-one without the presence of strangers. Locations were selected at community chat rooms in Guiyang City. Before interviews, participants were informed of the research objectives and completed informed consent forms. Strict adherence to voluntary participation and confidentiality principles ensured interview data were used solely for this study, with no disclosure of personal information. All interviews were audio-recorded, lasting between 20 and 40 min, with careful observation of participants’ nonverbal behaviors. Following each interview, participants verified the completeness and accuracy of the recorded data, with revisions and additions made promptly.

### 2.5 Data analysis

Within 24 h of concluding each interview, transcribe the audio recordings into text and import them into Nvivo 12.0 software. Two researchers independently organized and analyzed the data using Colaizzi’s 7-step analysis method, following these specific steps: (1) Repeatedly listen to interview recordings and carefully review transcripts to uncover participants’ underlying thoughts and experiences from their perspective; (2) Identify statements and words with deep meaning within participants’ experiences; (3) Code frequently occurring information that reflects participants’ viewpoints; (4) After coding, categorize and extract thematic concepts; (5) Integrate themes to more fully express participants’ authentic emotions; (6) Group similar viewpoints to refine themes; (7) Finally, present the extracted themes to interviewees for verification, ensuring they accurately reflect their genuine thoughts. Two researchers independently analyzed and coded the textual data, comparing results to synthesize themes. Disagreements were resolved through discussion within the research team.

### 2.6 Rigour and reflexivity

Before formal interviews, the researcher engaged in extensive reading of qualitative research literature and texts, accessed relevant qualitative research courses for systematic training, and mastered the application techniques of qualitative inquiry. The interview guide was designed based on research objectives and revised through iterative discussions by the research team. Before data collection, the researcher provided participants with a comprehensive debriefing to secure their active cooperation. During interviews, communication techniques such as probing questions, feedback, and confirmation were used to ensure that participants’ statements remained focused on the research objectives. Leading language was strictly avoided to prevent researcher bias from influencing participants’ perspectives. Interview data were independently transcribed and analyzed by two researchers, and the extracted themes underwent member checking—returning them to participants for verification to confirm whether the themes accurately reflected their genuine experiences. This process ensured the trustworthiness and validity of the qualitative findings.

## 3 Findings

NVivo software-assisted manual coding, which involved analyzing and categorizing data to form three themes and nine subthemes.

### 3.1 Theme 1: inadequate adaptability of external support systems

#### 3.1.1 Structural gaps in community-based home care policies and evaluation frameworks

The current assessment system within older adults’ care policies has numerous shortcomings, making it challenging to accurately cover older adults with mild cognitive impairment. This results in policies failing to meet the actual needs of this group during implementation (12).

P5: “The current disability assessment system for the older adults is completely unsuitable for someone like my father with mild cognitive impairment. Although he can manage daily living activities independently, his memory has deteriorated significantly. He requires constant reminders and guidance in his daily routine. Yet, because he does not meet the criteria for significant disability in the assessment, he cannot access certain services or receive relevant subsidies. This puts a lot of pressure on us.” P9: “Most existing disability assessment programs target individuals with pronounced disabilities. My mother, who has mild cognitive impairment, only exhibits prominent issues in cognition and mental state, making her ineligible for appropriate policy benefits. “P11:” Under the current assessment system, my father is simply categorized as having basic self-care abilities. Yet he has mild cognitive impairment, and we repeatedly hit roadblocks when applying for relevant policy support.”

#### 3.1.2 Shortages in caregiving knowledge and skills provision

After an older adult with mild cognitive impairment develops the condition, family caregivers face caregiving challenges and yearn to acquire professional caregiving knowledge and skills to better care for older people and handle various situations (13).

P6: “Her current condition leaves me helplessly anxious. I do not even know how to communicate effectively with her, I hope someone can teach me quickly.” P8: “My mom keeps forgetting things. I want to help her improve, but I have no idea where to start.” P14: “After my father was diagnosed with mild cognitive impairment, I’ve been learning about the condition, but I’m still unsure how to help him with rehabilitation exercises. It’s really frustrating. “P15:” I’m with my mother every day. Since her cognitive decline, her habits have changed, and I do not know how to guide her back to normal. I desperately need professional caregiving skills and knowledge.”

#### 3.1.3 Sustainability challenges in financial support

Caregivers bear a heavy financial burden, as professional nursing services are costly, and expenses for older adults’ care, rehabilitation training, and other needs continue to rise (14).

P16: “Shouldering the responsibility of caring for the older adults alone is overwhelming. Even a modest government subsidy would provide some financial relief.” P17: Just buying medication and rehabilitation therapy for my older adults relative costs thousands each month. My salary is fixed, and now I have to budget every household expense carefully. Take last month, for example—utility bills plus my relative’s care fees ate up more than half my paycheck. We’re barely scraping by.” P18: Lately, Dad’s cognitive decline seems worse. He keeps wandering around in the middle of the night. Worried he’ll fall and hurt himself, I bought a monitoring device—another expense. It’s not a significant illness, but all these little costs add up and suddenly we are over budget again.”

### 3.2 Theme 2: dual social and emotional burdens faced by caregivers

#### 3.2.1 Disruption of social networks and isolation

Caregivers often reduce social interactions due to the social withdrawal of older people, conceal caregiving pressures at work, and worry about hindering their career advancement. Simultaneously, fearing that community activities older adults’ pose older people’s condition, they restrict their social participation (15).

P5: “I used to chat with neighbors often, but now I barely have time to greet them. Plus, since my dad sometimes acts strangely, I feel embarrassed to invi “e neig” ors over.” P19: “At work, when colleagues occasionally discuss their older adults’ parents, I mention my father’s situation. If my superiors or coworkers learned I spent significant time caring for my sick father, I fear it could jeopardize my career advancement. They might perceive me as distracted and unable to fully “commit to my job.” P20: When community events are organized, I hesitate to let my mom participate, fearing she might forget things or say something inappropriate, which could reveal her condition to other older adults and their families. Last time, during a simple knowledge quiz, she answered incorrectly. I worried people nearby would notice her cognitive issues, fearing gossip and negative repercussions.”

#### 3.2.2 Accumulation of negative emotional experiences and burnout

Older adults individuals with mild cognitive impairment become increasingly prone to sadness and anxiety as their condition progresses. These negative emotions also affect the mental state of family caregivers, who must devote even more energy to providing care (16).

P1: “I once found my dad crying alone. When I asked what was wrong, he would not say anything for a long time. I’m so busy with work every day, and no one helps me. Caring for him is really stressful. Seeing him like that honestly makes me both angry and worried. “P3:” My husband can still cook and shop for groceries on his own. He used to love keeping the house tidy, but now it’s always messy. Or sometimes I clean up, and he cannot find things for ages, messing it all up again. I have to clean it all over. It’s impossible not to get annoyed.” P5: My dad refuses to believe he’s sick. Every time I ask him to come to the hospital, he gets angry and refuses to go. It’s really frustrating to see sometimes. “P7:” My husband gets emotional over the slightest thing lately, saying he’s useless. It makes me feel sad too.”

#### 3.2.3 Moral conflicts over family perceptions and responsibility allocation

In the care of older adults individuals with mild cognitive impairment, severe cognitive biases often exist within families and among relatives. The attribution of caregiving responsibilities frequently sparks moral disputes, straining family relationships. This places immense pressure on primary caregivers and hinders older people from receiving proper care (17).

P5: “My father’s mild cognitive impairment has severely impacted his daily life. He often forgets to turn off the stove while cooking and gets lost when leaving home. But my wife insists it’s not that serious and says I’m overreacting. “P9:” Since my mother’s diagnosis, her mood has become highly unstable—she’s often anxious and prone to outbursts. But my brother dismisses this as a normal emotional fluctuation in older people. He refuses to pay for professional caregivers or help me with her care. I feel utterly helpless. When I suggest a nursing home, the family objects.” P11: My dad needs supervision due to mild cognitive impairment, but other relatives insist it’s unnecessary to send him to a professional facility. They accuse me of shirking responsibility. They have no idea how hard it is for me or what my dad’s real condition is. They gossip behind my back, calling me heartless for abandoning an older adults person, but they never consider how difficult it is for me to care for him alone.”

### 3.3 Theme 3: obstacles in smart older adults’ care technology use

#### 3.3.1 Privacy concerns from smart monitoring systems

As innovative monitoring systems are increasingly used to ensure the safety of older adults individuals with mild cognitive impairment, privacy concerns have become more prominent, raising worries among family members (18).

P5: “The community installed surveillance to prevent my father, who has mild cognitive impairment, from getting lost. But the surveillance can directly capture the living room at home, and my daily activities with my family are all recorded, making me feel like there’s no privacy.” P8: “I installed smart surveillance in my mother’s room to monitor her condition at any time. But when my mother changes clothes or rests, I feel uncomfortable watching these private scenes as her son.” P11: I installed smart cameras to prevent accidents involving my father. However, once I discovered a connection issue with the cameras, I worried that others might be able to see the inside of my home. This risk of privacy leakage makes me very uneasy.”

#### 3.3.2 The digital divide

In the practical application of innovative older adults’ care systems, the complexity of operations leaves caregivers feeling overwhelmed, creating a digital divide that is difficult to bridge (19).

P5: “The community provided my father with smart older adults’ care devices, claiming they could monitor his health in real-time. However, the device’s interface design is extremely complex. My father has mild cognitive impairment, so it’s no surprise he cannot figure it out.” I studied it for a long time, but the various settings and function switches still confused me, and I even found it difficult to use.” P9: “To facilitate caring for an older adult family member with mild cognitive impairment, I downloaded a community smart older adults’ care app. However, the app’s functional layout is disorganized, and the operational processes are cumbersome. Every time I use it, I have to be extremely careful, fearing I might click the wrong option. Often, I’d rather not use it to quickly resolve issues and call the relevant service staff directly, which completely defeats the purpose of the supposed convenience of smart older adults’ care. “P11: The smart care system at the community’s day care center is supposed to provide better services for older adults like my father. However, when I tried it, I kept making mistakes, let alone my father. Now we can only rely on staff to manually operate it. The so-called smart older adults’ care system has not been effective at all.”

#### 3.3.3 Lack of emotional connectivity in human-computer interaction

In older adults’ care services, while human-machine interaction devices have been introduced to assist with caregiving, their limited emotional engagement makes it challenging to truly meet the emotional needs of older adults with mild cognitive impairment, and they often fail to alleviate the caregiving burden on caregivers (20).

P5: I applied for a smart companion robot service, hoping it could keep my father entertained. However, every time my father interacts with it, it responds mechanically. Now my father still hopes I can spend more time with him; the robot cannot replace me.” P9: “The community gave my mother a smart voice assistant to remind her of daily matters. But when my mother is in a low mood, the assistant does not notice and continues to remind her according to the program, offering no emotional comfort.” P11: “The nursing home arranged for my father to use smart care devices to monitor his health. However, when my father becomes confused, he fears the devices’ cold operation and mechanical voice (Table 2).”

**Table 2:** Open coding.

Level 1 code (subject)	Secondary code (sub-theme)	Level 3 coding (supported by primary data)
1. Inadequate adaptability of external support systems	1.1. Structural gaps in community-based home care policies and evaluation frameworks	1.1.1. Current disability assessments lack cognitive dimension considerations (P5, P9, P11); 1.1.2. Mild cognitive impairment does not meet criteria, preventing access to service subsidies (P5, P9, P11); 1.1.3. Policy applications face obstacles, placing heavy burdens on families (P5, P11).
	1.2. Shortages in caregiving knowledge and skills provision	1.2.1. Lack of communication methods with older adults individuals with cognitive impairment (P6); 1.2.2. Inability to conduct memory rehabilitation training (P8, P14); 1.2.3. Inability to guide older adults individuals in adjusting their lifestyle habits (P15).
	1.3. Sustainability challenges in financial support	1.3.1. Medication and rehabilitation costs are high and ongoing (P17); 1.3.2. Care equipment adds additional expenses (P18); 1.3.3. Without subsidy support, household finances are strained (P16, P17).
2. Dual social and emotional burdens faced by caregivers	2.1. Disruption of social networks and isolation	2.1.1. Reduced neighborhood social interactions due to older adults individuals’ unusual behaviors (P5); 2.1.2. Working caregivers conceal stress, fearing career repercussions (P19); 2.1.3. Restricting older adults’ community activities to avoid gossip (P20).
	2.2. Accumulation of negative emotional experiences and burnout	2.2.1. Older adults individuals’ non-compliance with treatment exacerbates physical and mental exhaustion (P1, P5); 2.2.2. Changes in daily routines trigger anxiety among the older adults (P3, P7); 2.2.3. Pressure with no outlet for release (P1).
	2.3. Moral conflicts over family perceptions and responsibility allocation	2.3.1. Family members hold inconsistent perceptions of the severity of the illness (P5, P9); 2.3.2. Relatives refuse to assume responsibility and reject institutional care (P9, P11); 2.3.3. Caregivers are accused of “shirking responsibility” and bear moral pressure (P11).
3. Obstacles in smart older adults’ care technology use	3.1. Privacy concerns from smart monitoring systems	3.1.1. Community surveillance covers residential living areas (P5); 3.1.2. Home surveillance involves private scenes of older adults individuals, raising ethical concerns (P8); 3.1.3. Equipment malfunctions spark worries about privacy breaches (P11).
	3.2. The digital divide	3.2.1. The device interface is complex and difficult for family members to operate (P5, P11); 3.2.2. The smart older adults’ care app features are disorganized and cumbersome (P9); 3.2.3. The intelligent system is prone to errors and relies heavily on manual intervention (P11).
	3.3 Lack of emotional connectivity in human-computer interaction	3.3.1. Intelligent robots offer mechanical responses, failing to meet emotional needs (P5); 3.3.2. Voice assistants lack emotional perception and comfort functions (P9); 3.3.3. Cold device operation triggers fear in the older adults (P11).

## 4 Discussion

### 4.1 Redesign the evaluation metrics system, enhance care skills empowerment, and optimize the provision of targeted services

This study indicates that the existing disability assessment system for older people, which focuses on individuals with pronounced disability, results in older adults individuals with mild cognitive impairment (MCI)—despite exhibiting memory decline and requiring prompting and guidance—being unable to access policy support and service subsidies because they do not meet traditional disability criteria. These findings align with the conclusions of Zhao et al. (21). This suggests that policy-level efforts should reconstruct the assessment logic by incorporating cognitive-related items such as memory function and communication ability into existing evaluation metrics, while simultaneously refining specialized subsidy policies (22). Concurrently, the findings reveal that the supply gap in caregiving knowledge and skills within family care settings further amplifies caregiving challenges. Caregivers commonly express feelings of helplessness when confronting issues like communication barriers, memory decline, and lifestyle changes in older people, yet lack effective channels to access professional guidance. This aligns with findings from Lobanov-Rostovsky et al. (23). A caregiving knowledge and skills supply system could be established through a combination of approaches, including offline lectures at primary healthcare institutions, online micro-videos and consultation channels, and caregiver mutual-aid communities (24). Research indicates caregivers also face persistent financial strain, aligning with Han et al.’s findings (25). Addressing this requires integrating medical, social, and public welfare resources to provide low-cost rehabilitation services and donate care equipment to struggling families, while exploring mechanisms to bridge commercial insurance with long-term care insurance (26). Among the 20 caregivers in this study, 14 were married and six were divorced. The majority were middle-aged individuals aged 35–55, predominantly residing in urban areas. Educational levels ranged from elementary school to a bachelor’s degree. Caregiving relationships were primarily parent–child, with a minority involving spouses or other family members. A notable proportion of caregivers resided in rural areas and had lower educational attainment. The diverse characteristics of the caregiver population indicate that policy and service provision must be grounded in clear support classifications and prioritization (27). Policies and service provision should be refined based on differences in caregivers’ age, educational attainment, marital status, and other factors. First, identify which support gaps most significantly impact caregivers’ burdens, prioritize enhanced financial subsidies for divorced caregivers, focus on in-person skills training courses for low-educated rural caregivers, and supplement emotional support resources for middle-aged caregivers, then develop differentiated strategies for distinct groups to address their diverse needs systematically (22).

### 4.2 Addressing the emotional challenges faced by family caregivers

The findings of this study indicate that caring for older adults individuals with mild cognitive impairment is a long-term process. Faced with increasingly unfamiliar behavioral patterns and cognitive distortions, caregivers become trapped in fear of an uncertain future, aligning with the results of Kim et al. (28). Simultaneously, the lack of professional caregiving guidance leaves caregivers feeling isolated and helpless when confronting the unique needs of older people. Misconceptions about cognitive impairment among those around them prevent caregivers from receiving the understanding and recognition their efforts deserve, consistent with the conclusions of Feng et al. (29). Additionally, negative emotions may lead to deviations in caregiving behavior. Prolonged fatigue and stress can cause caregivers to lose patience at certain moments, leading to overreactions to the older adult’s repetitive behaviors, which aligns with the findings of Pinyopornpanish et al. (30). To help caregivers navigate emotional challenges, communities should offer professional psychological counseling programs and regularly organize caregiver support groups. These gatherings provide opportunities to voice inner struggles and confusion, share experiences and insights, and alleviate emotional strain through mutual support. Simultaneously, enhanced training for caregivers is essential to improve their nursing skills and ability to manage emotional issues in older people, thereby boosting their confidence and sense of control in caregiving. Family members should also strengthen communication and collaboration to share caregiving responsibilities (31). Additionally, caregivers themselves are advised to learn emotional self-regulation through appropriate exercise, leisure activities, or hobbies to divert attention and maintain physical and mental balance and health (32).

### 4.3 Confronting the practical challenges of smart older adults’ care services

This study reveals that innovative older adults’ care services continue to face multiple challenges in their practical application. While existing smart monitoring devices can ensure real-time safety for older adults, they suffer from issues such as an unlimited monitoring scope, non-standardized data storage, and a high risk of privacy breaches. This phenomenon aligns with the findings of Zhu et al. (33). Consequently, some families resist smart older adults’ care devices due to privacy concerns, even abandoning their use, which severely hinders service promotion and implementation outcomes—a finding consistent with Ma B et al.’s research (34). Therefore, it is recommended to coordinate efforts across three dimensions: technology, institutional frameworks, and needs alignment. Technologically, ensure data encryption; institutionally, define monitoring boundaries to exclude private areas like bedrooms and bathrooms while refining informed consent procedures; and at the needs level, conduct in-home interviews and surveys to match caregiver requirements precisely, offering customizable options such as adjustable data retention periods and temporary monitoring deactivation to achieve a dynamic balance between security and privacy (35). Concurrently, this study reveals that most current smart older adults’ care devices and systems suffer from complex interface designs, redundant functions, and cumbersome operational workflows, lacking age-friendly considerations. Given that family caregivers are predominantly middle-aged or older adults individuals with relatively weak digital literacy, they struggle to quickly master usage methods, creating a significant digital divide (36). This undermines the intended convenience of innovative eldercare services. Some caregivers abandon smart devices due to operational difficulties, reverting to traditional care models—a finding consistent with Wang X et al.’s research (37). To address this, optimization should focus on three areas: design, training, and technical support. Design aspects should incorporate age-friendly features, such as large icons, high contrast, and voice guidance, to simplify core operations (e.g., emergency calls, data viewing) to three steps or fewer. Establish a tiered training system that offers one-on-one, hands-on instruction for family caregivers, alongside simplified manuals and video tutorials. Develop remote assistance features that enable caregivers to instantly call technicians for remote guidance or hands-on support when encountering operational challenges, thereby comprehensively lowering usage barriers (38). This study also reveals that existing human-machine interaction devices predominantly provide mechanical responses, lacking emotional awareness, and fail to meet the emotional needs of older adults. This limitation prevents innovative older adults’ care services from giving psychological support, potentially exacerbating loneliness due to emotional interaction deficits—consistent with findings by Lou and Liu (39). Therefore, deep integration of technology and humanistic elements is essential. AI interaction technology should be upgraded to recognize older adults’ emotional states through voice tone and facial expression recognition, dynamically adjusting responses—such as offering proactive comfort or playing soothing music during low moods (40).

## 5 Conclusion

This study, based on semi-structured interviews, reveals that family caregivers of older adults with mild cognitive impairment (MCI) confront multifaceted caregiving challenges. Challenges, including poor demand alignment, inflexible service delivery models, and inadequate integration of medical and geriatric care services, plague community-based home care services. Additionally, family caregivers generally lack systemic social support, enduring pressures such as social isolation, skill deficits, economic strain, and emotional distress. Concurrently, intelligent older adults’ care services demonstrate limited practical efficacy due to privacy infringements, operational intricacies, and emotional interaction deficits. It is recommended that relevant authorities conduct precise needs assessments, integrate medical and caregiving resources, optimize the content of community-based home care services, and promote the integration of smart older adults’ care services with humanistic care principles to enhance the quality of older adults’ care services in China comprehensively. This approach necessitates aligning service design with the dynamic needs of MCI patients, establishing standardized protocols for privacy-protective smart monitoring, and embedding emotional intelligence into technological innovations to bridge the gap between functional care and psychosocial support.

## Data Availability

The raw data supporting the conclusions of this article will be made available by the authors, without undue reservation.
